# Timescale analyses of fluctuations in coexisting populations of a native and invasive tree squirrel

**DOI:** 10.1002/ece3.8779

**Published:** 2022-04-06

**Authors:** Robert A. Desharnais, Alan E. Muchlinski, Janel L. Ortiz, Ruby I. Alvidrez, Brian P. Gatza

**Affiliations:** ^1^ Department of Biological Sciences California State University at Los Angeles Los Angeles California USA; ^2^ Center for Excellence in Mathematics and Science Teaching California State Polytechnic University at Pomona Pomona California USA

**Keywords:** climate change, fox squirrel, invasive species, population timescales, spectral analysis, Western gray squirrel

## Abstract

1. Competition from invasive species is an increasing threat to biodiversity. In Southern California, the western gray squirrel (*Sciurus griseus*, WGS) is facing competition from the fox squirrel (*Sciurus niger*, FS), an invasive congener.
2. We used spectral methods to analyze 140 consecutive monthly censuses of WGS and FS within a 11.3 ha section of the California Botanic Garden. Variation in the numbers for both species and their synchrony was distributed across long timescales (>15 months).
3. After filtering out annual changes, concurrent mean monthly temperatures from nearby Ontario Airport yielded a spectrum with a large semi‐annual peak and significant spectral power at long timescales (>28 months). The cospectrum between WGS numbers and temperature revealed a significant negative correlation at long timescales (>35 months). Cospectra also revealed significant negative correlations with temperature at a six‐month timescale for both WGS and FS.
4. Simulations from a model of two competing species indicate that the risk of extinction for the weaker competitor increases quickly as environmental noise shifts from short to long timescales.
5. We analyzed the timescales of fluctuations in detrended mean annual temperatures for the time period 1915–2014 from 1218 locations across the continental USA. In the last two decades, significant shifts from short to long timescales have occurred, from <3 years to 4–6 years.
6. Our results indicate that (i) population fluctuations in co‐occurring native and invasive tree squirrels are synchronous, occur over long timescales, and may be driven by fluctuations in environmental conditions; (ii) long timescale population fluctuations increase the risk of extinction in competing species, especially for the inferior competitor; and (iii) the timescales of interannual environmental fluctuations may be increasing from recent historical values. These results have broad implications for the impact of climate change on the maintenance of biodiversity.

## Graphical abstract

This study documents long timescale variation in natural populations of conservation interest, shows that long timescale variation can accelerate the loss of species diversity, and provides evidence that the timescales of environmental fluctuations have increased in recent times. These results have broad implications for the impact of climate change on the maintenance of biodiversity.

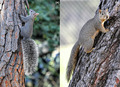

## 1 INTRODUCTION

Competition from non‐native, invasive species is an increasing threat to the biodiversity of native species in a globalized world. Invasive species are often considered one of the most important threats to ecological function and a top driver of species extinctions (Dueñas et al., 2021; Flory & Lockwood, 2020). The presence of invasive species can alter animal communities, trigger trophic cascades, displace native species, and even lead to hybridizations with similar or related species (Doody et al., 2017; Huxel, 1999). The ability to be more competitive over limited resources is one of the characteristics that enables invasive species to be successful. In addition, they are often characterized by having life history traits with colonizer characteristics as follows: short generation times, high reproduction rates, and fast growth rates (Sakai et al., 2001). With this competitive edge, they can invade and displace native species.

**FIGURE 1:**
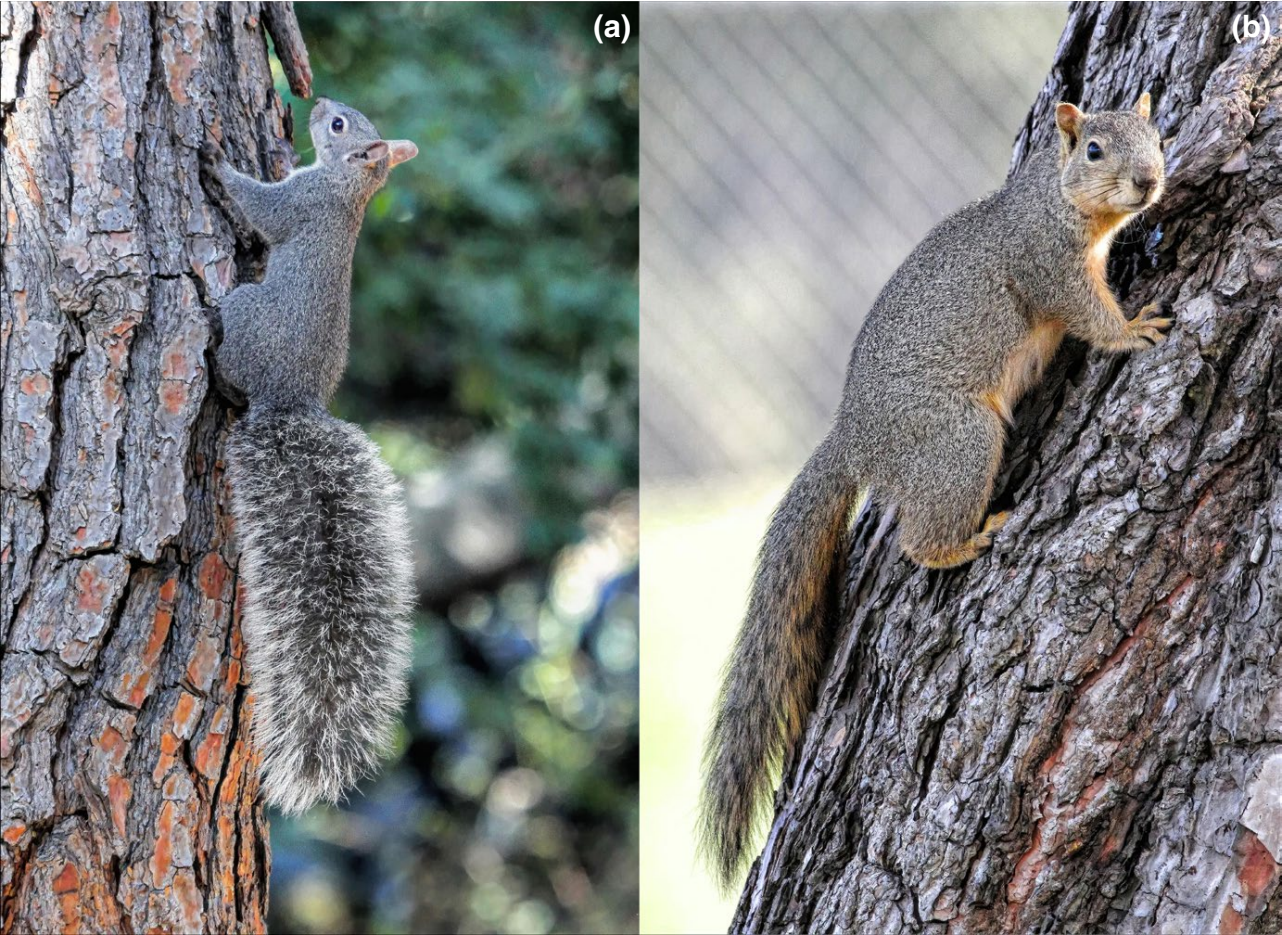
Photographs of the two diurnally active tree squirrels that are present in Southern California: (a) the native Western Gray Squirrel, *Sciurus griseus* and (b) the non‐native Fox Squirrel, *Sciurus niger*. The Fox Squirrel has replaced the Western Gray Squirrel in some habitats, while the two species coexist in other habitats. Photographs by Alan Muchlinski

An example where a native species is threatened in some habitats by competition from an invasive species occurs in Southern California, where the western gray squirrel (*Sciurus griseus*, WGS, Figure 1a) is facing increasing competition from the fox squirrel (*Sciurus niger*, FS, Figure 1b), a non‐native, invasive congener. WGSs are native to the western coast of North America with a historical distribution extending from central Washington to Baja California (Carraway & Verts, 1994; Escobar‐Flores et al., 2011). Populations of WGSs have been declining in areas of Washington, Oregon, and California (Cooper, 2013; Cooper & Muchlinski, 2015; Muchlinski et al., 2009; Stuart, 2012). In Washington, they are listed as a state‐threatened species (Linders & Stinson, 2007), while in Oregon they are an Oregon Conservation Strategy Species (Oregon Department of Fish & Wildlife, 2016). While there have been only a few studies regarding populations of WGSs in California, there is a noticeable trend in the decline of these squirrels in areas below an elevation of 457 m (Cooper, 2013; Cooper & Muchlinski, 2015). As of now, the WGS does not have special conservation status in California.

The FS has a historical native range in the eastern and central United States and the southern prairie provinces of Canada, south of approximately 48ºN latitude (Koprowski, 1994), where they are known to live in forests, woodlands, agricultural landscapes, and urban areas (Kleiman et al., 2004). Through both natural and human‐assisted range expansion, the FS is now common in many areas west of its historical range (iNaturalist accessed 24 July 2021, https://www.inaturalist.org/taxa/46020‐Sciurus‐niger). Fox squirrels have been introduced or have expanded their range into Arizona, California, Colorado, Idaho, Montana, New Mexico, Oregon, Utah, Washington, and Wyoming (Brady et al., 2017; Flyger & Gates, 1982; Jordan & Hammerson, 1996; Koprowski, 1994; Steele & Koprowski, 2001; Wolf & Roest, 1971).

Fox squirrels have dispersed from original points of introduction through natural dispersal and through intentional movement of animals by humans (Frey & Campbell, 1997; Geluso, 2004; King et al., 2010). Since the original introduction to Los Angeles County (Becker & Kimball, 1947), the FS has expanded its range at a rate of 1.60 to 3.00 km/year in heavily suburbanized areas of Southern California (Garcia & Muchlinski, 2017). Although the FS has generally remained restricted to areas of human habitation, with continued range expansion the FS has become sympatric in some isolated suburban habitat fragments and in certain foothill areas with the native WGS (Hoefler & Harris, 1990).

FSs may compete with native WGSs for resources such as nesting sites and food, and the FS has replaced the WGS within certain habitats in Southern California (Cooper & Muchlinski, 2015; Muchlinski et al., 2009). Los Angeles County can be considered an ideal location for invasion by the FS given the mild Mediterranean climate and year‐round food supply offered by exotic plant species, accompanied by the absence of the native WGS throughout much of the Los Angeles Basin. The FS is both morphologically, ecologically, and behaviorally similar to this native species; thus, these overlaps in form, function, activity, and presence provide a situation where interactions between the two species can be studied (Ortiz, 2021).

Many factors can influence population persistence, but one that has received comparatively less attention is the timescale of environmental and population fluctuations. In the current study, we investigated the effects of timescales using three approaches. First, we applied spectral analyses to examine the timescale distribution of the variance and covariance of WGS, FS, and weather time series. Second, we conducted model simulations to examine the implications of changes in the timescale of environmental fluctuations on the coexistence of a native species facing competition from an invasive species. Third, we used spectral and wavelet methods to examine the long‐term changes in the timescale distribution of climate data.

By analogy with the spectrum of visible light, time series fluctuations that occur over long timescales are referred to as having a *red spectrum* and those occurring over short timescales as having a *blue spectrum* (Lawton, 1988). These are distinguished from *white noise* random fluctuations, which have no serial autocorrelations. In general, theoretical analyses from single‐species discrete‐time unstructured population models suggest that the response to colored environmental noise depends on the type of population dynamics. Deterministic models with stable equilibria can exhibit *undercompensatory* dynamics, where the population approaches equilibrium monotonically, or *overcompensatory* dynamics, where the approach to equilibrium exhibits damped oscillations. Many studies have shown that reddened environmental spectra increase extinction risk for undercompensatory populations and blue spectra increase extinction risk for overcompensatory populations (Danielian, 2016; García‐Carreras & Reuman, 2011; Mustin et al., 2013; Petchey et al., 1997; Ripa & Heino, 1999; Ripa & Lundberg, 1996; Ruokolainen et al., 2009; Schwager et al., 2006). While some studies reached different conclusions (Heino, 1998; Heino et al., 2000), Schwager et al. (2006) showed that these contrasting results depend on the modeling details and a consideration of the likelihood of catastrophic events. In their simulations of three competing species, Ruokolainen and Fowler (2008) found that extinction risk increased with reddened environmental noise when species responded independently to the environment but decreased when there was a strong correlation between species‐specific responses. On the empirical side, Pimm and Redfearn (1988) looked at 100 time series from insects, birds, and mammals and found that the variance of the population fluctuations increased with the window of time used in the calculation, suggesting that these populations have red spectra. García‐Carreras and Reuman (2011) analyzed the dynamics of 147 animal populations and climate data for the population locations and found a positive correlation between the biotic and climatic spectral exponents (a measure of spectral color), with most spectra being red‐shifted. Inchausti and Halley (2003) directly examined the relationship between population variability and quasi‐extinction time (measured as the time required to observe a 90% decline of population abundance) for a large set of data comprised of 554 populations for 123 animal species that were censused for more than 30 years. The results showed that the quasi‐extinction time was shorter for populations having higher temporal variability and redder dynamics. In a laboratory microcosm experiment, Fey and Wieczynski (2017) looked at how the autocorrelation in thermal warming affected the ability of a non‐native cladoceran, *Daphnia lumholtzi*, to establish itself in the presence of a native congener, *D*. *pulex*. The non‐native species was able to attain significantly higher population densities in the treatment with an autocorrelated warming regime relative to the treatment with uncorrelated warming but the same mean temperature and the unwarmed control. Although, in all three treatments, *D*. *lumholtzi* went extinct by the end of the experiment, their results demonstrated that the timescale of environmental fluctuations can impact the ability of an invasive species to establish itself in the presence of a native competitor.

Spectral methods are a powerful tool for characterizing the timescales of fluctuations in a time series (Brillinger, 2001). A univariate time series can be transformed into a *power spectrum*, which describes the distribution of the variance of the time series at different frequencies. The sum of the spectral powers across frequencies is proportional to the total variance of the time series. If the time series is multivariate, in addition to the spectra, there are also cross‐spectra for each pair of time series variables. The cross‐spectrum is a complex‐valued function of frequency. The real part is the *cospectrum*, which describes the distribution of the in‐phase covariance between the time series at different frequencies, and the imaginary part is the *quadrature spectrum*, which is a phase‐shifted covariance. The sum of the cospectral powers across frequencies is proportional to the total covariance of the two time series. The cospectrum can also be viewed as the distribution of the correlation coefficient across frequencies. Since frequency, *f*, is the inverse of the period, the spectral and cospectral power provide information on the variance and correlation, respectively, at the timescale 1/*f*.

The color of a power spectrum can be characterized using a *spectral exponent* (García‐Carreras & Reuman, 2011; Vasseur & Yodzis, 2004). If *S_f_* is the power of the spectrum at frequency *f*, the spectral exponent can be computed as the slope of a least‐squares linear regression of log(*S_f_*) versus log(*f*). Negative spectral exponents are characteristic of spectra dominated by long timescale variation (red spectra), and positive values are indicative of short timescale variation (blue spectra). White noise spectra will have a spectral exponent of zero. When applied to environmental and population time series, spectral color allows one to better assess the risk of ecological extinction.

Wavelet analyses have been used in ecology to identify changes in the spectral distributions of population and environmental fluctuations over time (Cazelles et al., 2008). Whereas spectral analyses assume that the statistical properties of the time series do not vary with time, wavelet analysis can be applied to non‐stationary time series. A filtering function is applied to the time series signal to allow a local estimation of spectral characteristics of the signal at a point in time. The filtering function can be adjusted to look at different times and frequencies. The result is a two‐dimensional picture of the wavelet power as a function of frequency and time. Wavelets can be used, for example, to investigate the impact on ecological populations of climate regime shifts, such as the North Atlantic Oscillation (Sheppard et al., 2016), or changes in the timescale of environmental fluctuations due to climate change.

Global climate is undergoing rapid changes (Masson‐Delmotte et al., 2021). While the threats to biodiversity have focused mostly on increasing temperatures, it is feasible that disruptions to climate patterns may also affect the timescale of environmental fluctuations, and, if so, this may have ecological implications for population persistence. For example, García‐Carreras and Reuman (2011) analyzed detrended mean summer temperature time series from weather stations on six continents and found significant shifts to shorter timescales (blue shifts) in the spectral exponents for the years 1951–1990 compared with 1911–1950. Wang and Dillon (2014) analyzed annual global temperature cycles from 1975 to 2013 and found significant increases in the autocorrelation of temperature values (red shifts) in tropical and temperate regions. Di Cecco and Gouhier (2018) examined air temperature values predicted by 21 global circulation models under the business‐as‐usual scenario and found that spectral exponents were predicted to shift negatively to longer timescales from the years 1870 through 2090. For conservation purposes, it is important to gain a better understanding of how changes in climate may be associated with changes in the timescale of environmental fluctuations and how this may impact extinction risks for natural populations.

The objectives of the present study were (1) to evaluate, using spectral methods, the timescale of population fluctuations in a long time series (140 months) where the WGS and FS have coexisted together, (2) to determine the extent to which the timescale of the squirrel population fluctuations are determined by environmental factors, (3) to infer, using model simulations, how changes in the timescale of environmental fluctuations could impact the timescale of population fluctuations and the risk of extinction in a system of two competing species, and (4) to assess the extent to which the timescale of year‐to‐year environmental fluctuations around their trends is changing, possibly as a result of human impacts on climate, and to assess the implications of these results on the potential loss of native biodiversity.

## 2 MATERIALS AND METHODS

### 2.1 Collection of census data

We established three transect lines within a 11.3 ha section of the California Botanic Garden (CBG) in Claremont, CA, during October of 2009. We defined sampling points along transect lines at 40‐m intervals providing 35 viewpoints within the study area. Two researchers conducted a census along the transect lines once per month from October 2009 through May 2021. The researchers spent 3 minutes at each sampling point, with each researcher responsible for counting animals within a separate 180‐degree arc from the viewpoint. We began each monthly census at 0800 h and ended at approximately 1030 h. We switched the starting transect line for the monthly census between Line 1 and Line 3 on alternate months.

Researchers conducting each census were conservative in counting the number of squirrels observed, thereby giving an estimate of observable population size at a point in time. If there was any chance that a squirrel observed at a sampling point had been counted at a previous sampling point, that individual was not counted as a new observation unless the animal was obviously different from the animal previously observed (a juvenile instead of an adult or a male instead of a female, when gender could be assessed). Numbers may vary due to factors such as natality, mortality, dispersal, and activity levels, which could change due to seasonality or reproductive activity.

The four corners of the 11.3 ha study area were defined by the following GPS coordinates: SE 34.110262 & −117.714651, SW 34.110258 & −117.715921, NE 34.115883 & −117.714419, NW 34.115684 & −117.715891. CBG is a native California garden, meaning all plants are native to California, but not specifically Southern California. At the beginning of the study in 2009, the habitat within the study area included 1048 trees along with numerous shrubs and bushes. Of the trees, 31% of the species were deciduous, with 69% being non‐deciduous. Seventeen percent of the total trees were coniferous (83% not coniferous); 42% of all trees were in the genus *Quercus*; and 6% of all trees were in the genus *Pinus*. The composition of the study area did change over the time period of the censuses with the death and removal of several trees. Death of trees in the study area was due mainly to a prolonged drought within Southern California from 2011 through 2016.

### 2.2 Spectral analyses of census data

We used spectral methods to analyze the monthly census data. We used fast Fourier transforms to compute the raw spectra and cross‐spectrum of the bivariate time series. Computations were conducted using the spec.pgram algorithm from R modified to run in MATLAB. No trends were removed from the data prior to analysis. Since raw spectra and cross‐spectra are usually jagged, we applied two iterations of a window‐averaging smoothing Daniell kernel with spans of 5 and 7 data points, modified with clipped windows at the endpoints to preserve the number of data values. We divided the spectral powers by their sum across frequencies. This yielded a normalized spectral power plot for each species, which shows the distribution of variation across timescales. We used the real part of the cross‐spectrum to obtain a smoothed cospectral power plot for the covariance between the two species. We normalized the cospectrum so that its sum equals the correlation coefficient.

We conducted computations to detect significant (*p* < .05) peaks or valleys in the observed spectra for the null hypothesis that there is no frequency dependence in the variance and covariance of the time series fluctuations (i.e., independent “white noise” time series). We shuffled the temporal order of the bivariate time series by generating a random permutation of the integers 1 through *n*, where *n* = 140 is the number of monthly observations. We then used the permutation to reorder the bivariate monthly censuses of the two species. Next, we computed two smoothed normalized spectra and a smoothed normalized cospectrum in the same way as the correctly ordered data. We repeated this random reshuffling process 2000 times. For the spectra, which must be nonzero, we used the 95th percentile at each frequency to define one‐sided upper 95% confidence limits for the null hypothesis that there are no timescale components to the variance. For the cospectra, which can be positive and/or negative, we used the 2.5th and 97.5th percentile at each frequency to define two‐sided 95% confidence limits for the null hypothesis that there are no timescale components to the correlation. This method of generating the spectra preserves the time‐independent statistical properties of the two time series (means, variances, distribution, total correlation, etc.), while varying only the time‐dependence of the bivariate data values.

### 2.3 Analyses of weather data

We obtained weather data for Ontario Airport (ONT) from the Climate Data Online website of NOAA’s National Centers for Environmental Information (https://www.ncdc.noaa.gov/cdo‐web/). ONT is located about 12 km from the CBG and should be an accurate representation of the temperature profile of the study site. We focused on the reported “average monthly temperature,” which is computed by averaging the daily maximum and minimum temperatures for each month. We avoided rainfall totals because many months have zeros, which is a problem for spectral analyses, and much of the vegetation in the CBG is irrigated. We obtained a temperature time series for the same months as the census data and applied the same spectral methods to obtain a smoothed normalized power spectrum.

Since annual seasonal changes dominated the temperature time series, we used the MATLAB “band‐stop” function to attenuate cyclic components with periodicities in the range of 9–15 months. This produced a filtered time series with annual effects removed. We then produced a smoothed normalized spectrum for the filtered temperature time series. We also generated smoothed normalized cospectra between the filtered temperature time series and both the WGS and FS census time series. Using the methods described above, we obtained 95% confidence intervals for these spectra and cospectra.

### 2.4 Model simulations

We conducted model simulations to obtain a better understanding of the implications of timescale‐specific environmental variation on the dynamics of two competing species. We used the following discrete‐time version of the Lotka–Volterra competition equation:

(1) $$\begin{array}{c} N_{1}\left(t+1\right)=N_{1}\left(t\right)\exp\left(r_{1}\left(K_{1}‐N_{1}\left(t\right)‐\alpha N_{2}\left(t\right)\right)/K_{1}+\sigma_{1}\varepsilon_{1}\left(t\right)\right),\\ N_{2}\left(t+1\right)=N_{2}\left(t\right)\exp\left(r_{2}\left(K_{2}‐N_{2}\left(t\right)‐\beta N_{1}\left(t\right)\right)/K_{2}+\sigma_{2}\varepsilon_{2}\left(t\right)\right), \end{array}$$

where *r*_1_ and *r*_2_ are the intrinsic rates of population increase, *K*_1_ and *K*_2_ are the carrying capacities, and *α* and *β* are the competition coefficients for the two species. The variables *ε*_1_(*t*) and *ε*_2_(*t*) represent random environmental noise with a mean of zero and variance of .5. We used the coefficients σ_1_ and σ_2_ to scale the magnitude of the noise. For the purposes of discussion, species 1 will represent a native species and species 2 will represent an invasive species.

We introduced frequency‐specific biases into the noise variables using an algorithm devised by Chambers (1995). This method generates a multivariate random time series based on any specified theoretical spectral matrix that is a function of frequency. The diagonal elements of that matrix are the theoretical spectra (frequency decompositions of the variances), and the off‐diagonal elements are theoretical cross‐spectra (complex numbers). The real parts of the cross‐spectra are the theoretical cospectra (frequency decompositions of the covariances), and the complex parts are the quadrature spectra (frequency‐specific phase shifts). For the model (1), we used identical spectra that were linear functions of frequency for the two species. High‐frequency‐biased blue noise was represented with a linear spectrum that varied from a power of 0.0 for a frequency of *f* = 0.0 to a power of 1.0 for a frequency of *f* = 0.5 (maximum possible frequency). Low‐frequency‐biased red noise was represented with a linear spectrum that varied from a power of 1.0 for a frequency of *f* = 0.0 to a power of 0.0 for a frequency of *f* = 0.5. Unbiased white noise had a constant power of 0.5 across all frequencies. A gradual shift from blue to white to red noise was accomplished by varying the slope of the noise spectrum in 101 increments while keeping the average of the spectrum constant at 0.5. This produced a constant total variance of *ε*_1_(*t*) and *ε*_2_(*t*) equal to 0.5 while changing only its frequency‐specificity. For the covariance between the random variables *ε*_1_(*t*) and *ε*_2_(*t*), we used a cospectrum function that was equal to a constant fraction, 0.9, of the spectrum. This resulted in a frequency‐specific correlation of 0.9 across all frequencies. A high correlation was used since it was assumed that the native and invasive species are ecologically similar and occupy the same habitat. The quadrature spectrum was set to zero (no frequency‐specific phase shifts). To summarize, the timescales of the random environmental noise were varied from short (blue) to uniform (white) to long (red) with a frequency‐independent correlation in the effects of the noise on the growth of the two species.

In addition to the spectral frequency of the environmental noise, the simulation protocol also involved varying the competition coefficient *α*, which represents the intensity of the competitive effects of the invasive species on the native species. We set the value of the *α* to .25 (weak competition), .50 (moderate competition), and .75 (strong competition). We kept the competitive effects of the native species on the invasive species at a value of *β* = .25 while increasing *α* because we are interested in situations of concern to conservationists where an invasive species outcompetes the native species. We chose values for the intrinsic rate of increase *r*_1_ = *r*_2_ = .3 that are appropriate for tree squirrels of the genus *Sciurus* (Appendix A). The remaining model parameters had constant values of *K*_1_ = *K*_2_ = 50, and *σ*_1_ = *σ*_2_ = 0.75. For the assessment of extinction risk, when the population density of a species fell below 5% of its carrying capacity, we set it to zero. For simulations not involving extinction risk, the threshold was set to zero. We ran each simulation for 100 time steps.

For every set of parameter values and environmental noise color, we conducted 2000 replicate simulations. For blue, white, and red environmental noise, we computed smoothed normalized power spectra and cospectrum of the species and averaged these over replicates to see how the timescale for population fluctuations is affected by different colors of noise. To investigate gradual shifts in the effects of frequency‐biased environmental noise on the population spectra and probability of extinction, we chose a slope for the environmental spectra, varying the slopes in 101 gradual increments, beginning at blue noise (slope = 2) and ending at red noise (slope = −2). For each choice of the environmental spectra, we simulated the population trajectories of the two species, estimated the unsmoothed normalized population spectra, computed the two spectral exponents, and averaged them. In the same way, we computed average spectral exponents for the environmental noise realizations. We repeated these computations for each of the 2000 replicate simulations and computed an overall average for the spectral exponents of the populations and noise. To investigate the effects of frequency‐biased environmental noise on ecological persistence, the number of instances where each population went extinct was divided by 2000 to yield estimates of the extinction risks for both the native and the invasive species.

### 2.5 Analyses of climate data

We obtained climate data from the U.S. Historical Climatology Network (USHCN), which is freely available online (https://www.ncei.noaa.gov/products/land‐based‐station/us‐historical‐climatology‐network). We used version 2.5 of the monthly temperature records, which contains long‐term data from 1218 stations across the continental United States. Menne et al. (2009) describe the adjustments used to remove biases due to factors such as relocation of recording stations, changes in instrumentation, and urbanization. USHCN monthly average temperatures were computed as the average over the month of the daily maximum and daily minimum temperatures. The mean annual temperature for each year is the average of the 12 mean monthly temperatures. We used the mean temperatures for the 100‐year range from 1915 through 2014, the latter being the latest year available.

We looked at changes in the distribution of spectral exponents for the fluctuations in the mean annual temperatures. First, we broke the 100‐year range into four 25‐year spans. Next, we detrended the temperature time series for each 25‐year span by fitting a quadratic polynomial using least‐squares regression and computed the standardized residuals. Then, we computed an unsmoothed spectrum for each residual time series and estimated the spectral exponent as the slope of a linear regression of log(spectral power) versus log(frequency). Histograms were created with the 1218 spectral exponents (one per station) for each of the 25‐year time spans.

Although it would be tempting to analyze the changes in the spectral exponents using a repeated measures ANOVA, with stations as the subjects, spatial autocorrelations exist among stations that are in the same geographical proximity, inflating the Type I error rates. A solution to this problem was suggested by Clifford et al. (1989) and modified by Dutilleul (1993), which yields an “effective sample size” based on the spatial structure of the data. It is appropriate for paired observations distributed in space. We used the software package SAM (Spatial Analysis in Macroecology; Rangel et al., 2006) to compute effective sample sizes for the following three sets of paired data: [1915–1939] vs. [1940–1964], [1940–1964] vs. [1965–1989], and [1965–1989] vs. [1990–2014]. We conducted paired sample *t*‐tests for the spectral exponents from these three paired data sets and adjusted the standard errors for the test statistics and degrees of freedom for the statistical significance values using the effective sample sizes. We then applied a Bonferroni correction to account for the multiple comparisons.

We also conducted a mean field wavelet analysis on the 100‐year time series of mean annual temperatures. For each station, we detrended the time series using a quadratic polynomial and computed the standardized residuals. Next, we used the MATLAB continuous wavelet transform function “cwt” to compute wavelet powers for the residual time series using the analytic Morse filter (Olhede & Walden, 2002) with the default values of 3 for the symmetry parameter and 60 for the time‐bandwidth product. Lastly, we averaged the wavelet powers across all stations for each time–frequency combination. We chose the Morse wavelet because it is useful for analyzing signals with time‐varying amplitude and frequency. We investigated varying the symmetry and time‐bandwidth product parameters, but the results were not much different from what was obtained using the default values. We also used a Morlet wavelet which has equal variance in time and frequency, but, again, the results were like the Morse wavelet with default parameters. We experimented with cubic and quartic polynomials for detrending, but these gave mean field wavelets that were much like the one obtained with a quadratic function. Our mean field wavelet differs from the “wavelet mean field” defined by Sheppard et al. (2016), which was designed as an average measure of the time‐ and timescale synchrony for time series from different locations.

To identify wavelet powers that were statistically significant, we used the surrogate time series approach (Schreiber & Schmitz, 2000). We took a random permutation of the mean annual temperature time series for all stations in tandem and computed a mean field wavelet as described above. We repeated this process 2000 times and computed the upper 95th percentile of the wavelet powers for each combination of time and frequency. This provided a set of critical values for identifying “hot spots” on the mean field wavelet under the null hypothesis of no timescale dependence in the fluctuations of the mean annual temperature residuals around the trends.

## 3 RESULTS

### 3.1 Census data

**FIGURE 2:**
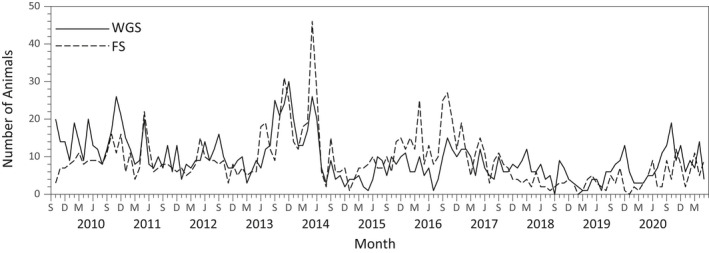
Monthly time series for numbers of WGS and FS at our study site from October 2009 through May 2021

Figure 2 shows the time series of monthly census values for the WGS and the FS. The large increase in census numbers during 2013 and 2014 corresponded with the production of a large acorn crop during the fall of 2013 (mean ± SE of 608.3 ± 120.1 g/m^2^ in a 1 m^2^ plot under each of six trees used to assess acorn production, Appendix B). Mean acorn production measured in the same 1 m^2^ plots during other years ranged from a low of 5.7 ± 2.7 g/m^2^ in 2014 to a high of 67.5 + 35.5 g/m^2^ in 2012. Availability of acorns appears to have a major impact on the number of WGSs and FSs in the CBG.

The fluctuations in census numbers show signs of synchrony. The estimated Pearson correlation coefficient in animal numbers is *R* = .581 which is statistically significant from zero (*p* = 5.20 × 10^−14^). The total variation in the numbers for each species and their synchrony seems to be distributed across different timescales. Long intervals can be seen where the numbers of both species are elevated and depressed (Figure 2). Superimposed on this long timescale variation are random short timescale fluctuations. We quantify this timescale component of variation with the spectral analyses in the next section.

### 3.2 Population spectra and cospectrum

**FIGURE 3:**
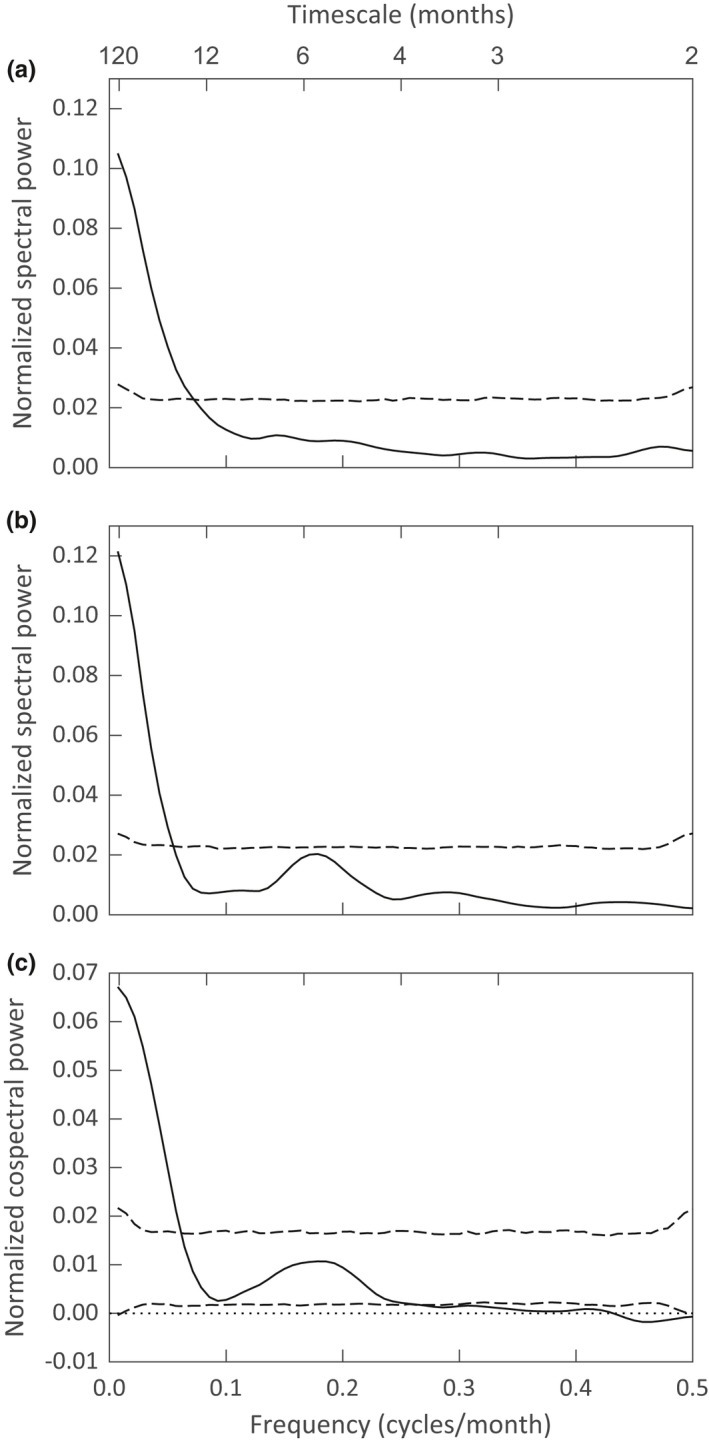
Smoothed normalized spectra for (a) the WGS and (b) the FS and the (c) smoothed normalized cospectrum between the two species. Dashed lines are 95% significance thresholds for the null hypothesis of no timescale dependence of the WGS‐FS paired observations

Figure 3a,b show the smoothed normalized spectra for the WGS and the FS. For both the WGS and the FS, the spectra suggest that the largest variation in numbers occurs at frequencies below 0.0833 which corresponds to a timescale of more than 12 months. The WGS spectrum crosses the upper significance threshold at timescale of around 15 months. The FS spectrum crosses the upper significance threshold at timescale of around 18 months. The spectrum for the FS shows a small peak at 6 months, but that peak is not statistically significant. Since the total variation remains constant across frequencies for the confidence bands from the randomly ordered data, the larger variation in WGS and FS at long timescales is compensated for by smaller variation at timescales of about 4 months or less.

The smoothed normalized cospectrum (Figure 3c) shows how the total correlation in population numbers between the two species is distributed across timescales. Covariance between WGS and FS is significantly biased toward long timescales, with a smaller nonsignificant peak at a timescale of around 6 months. The cospectrum crosses the upper significance threshold at timescale of about 18 months. The total correlation between the numbers of the WGS and FS is *R* = .581. Using the unsmoothed cospectrum, we can partition this total correlation by timescale intervals: *R*_1_ = .409 for >12 months, *R*_2_ = .162 for 4–12 months, and *R*_3_ = .010 for ≤4 months, where *R* = *R*_1_ + *R*_2_ + *R*_3_. Thus, 70% of the total correlation occurs at timescales exceeding one year. We can infer that population synchrony for these two species occurs mostly at long timescales.

### 3.3 Spectral analyses of weather data

**FIGURE 4:**
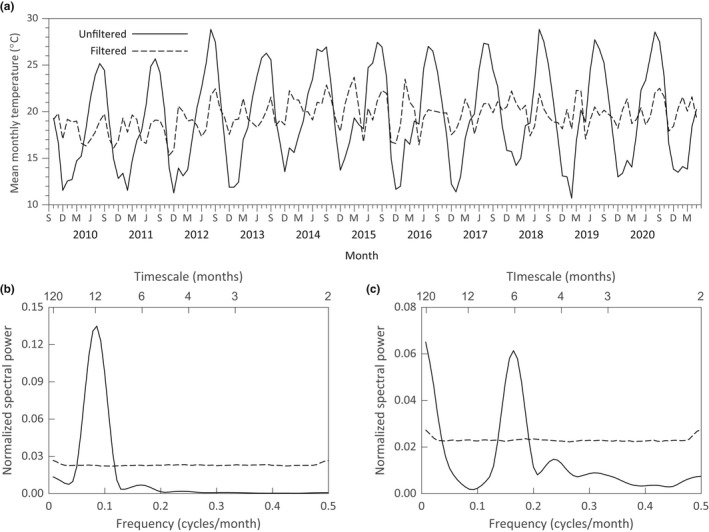
(a) Mean monthly temperature for the Ontario Airport (ONT) time series from October 2009 through May 2021. The solid line is for the recorded temperatures and the dashed line is the time series obtained after filtering out the annual cycle. Smoothed normalized spectra are shown for the (b) unfiltered and (c) filtered ONT temperature time series. Dashed lines in (b) and (c) are 95% significance thresholds for the null hypothesis of no timescale dependence in the ordering of the filtered and unfiltered data

Figure 4a shows the time series of mean monthly temperatures for Ontario Airport (ONT), which is 12 km from the study site. As one would expect, there is a strong seasonal component to these temperatures. Figure 4b shows the smoothed normalized spectrum for the mean monthly temperatures, which is dominated by a strong peak for the annual cycle. Since the squirrel spectra show no indication of an annual cycle (Figure 3), we applied a band‐stop filter to remove the annual cycle and plotted the resulting time series (Figure 4a, dashed line). The smoothed normalized spectrum for the filtered mean monthly temperatures appears in Figure 4c. There is a peak at low frequencies which crosses the upper threshold for statistical significance at a timescale of approximately 28 months and reaches a minimum at a timescale of 12 months. There is also a large spectral peak at 6 months.

**FIGURE 5:**
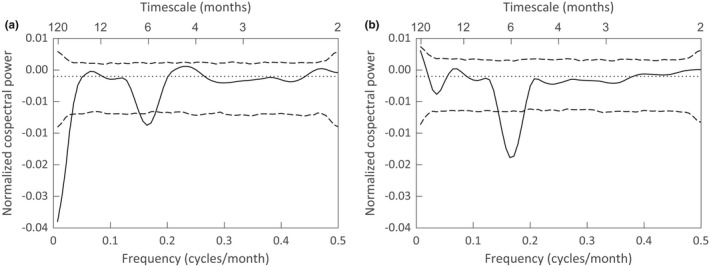
Smoothed normalized cospectra between the filtered Ontario Airport temperature time series and the census numbers for (a) the WGS and (b) the FS. Dashed lines in (a) and (b) are 95% significance thresholds for the null hypothesis of no timescale dependence in the ordering of the temperature, WGS, and FS data triplets

There is a negative correlation between the filtered temperature time series and the squirrel census data. For the WGS, the correlation is statistically significant (*R* = −.194, *p* = .022) and, as indicated by the smoothed normalized cospectrum (Figure 5a), is distributed at long timescales (>35 months) and at a timescale of 6 months. The correlation between the filtered temperature time series and the FS census data is also negative, but not statistically significant overall (*R* = −.146, *p* = .085). The cospectrum between the filtered temperature time series and FS census data shows a large significant peak at a 6‐month timescale (Figure 5b). These results suggest that the distribution of variation in the squirrels’ population fluctuations may be driven, in part, by fluctuations in weather and climate outside of the annual seasonal cycle.

### 3.4 Simulation results

**FIGURE 6:**
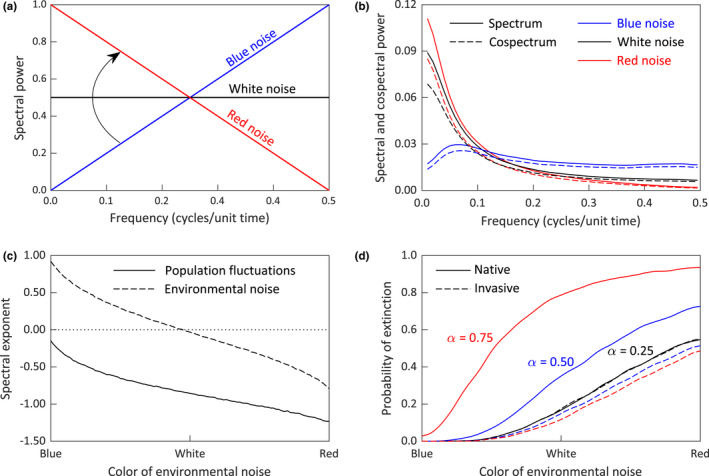
(a) Linear environmental spectra used for the model simulations. Each spectrum has the same variance, but a different distribution of variation over timescales. The arrow indicates how the spectra were gradually changed from blue noise, to white noise, to red noise for the simulations in panels (c) and (d). (b) Mean smoothed normalized spectra and cospectra for the populations with the blue, red, and white environmental noise shown in panel (a). Both competing populations had the same parameter values, so their spectra were identical. (c) Mean spectral exponents for the populations (solid line) and environmental noise (dashed line) with environmental noise color varied continuously from blue to white to red as shown in panel (a). More negative slopes indicate longer timescale fluctuations in population numbers. (d) Probabilities of extinction for the native species (solid lines) and invading species (dashed lines) for 2000 simulations of the model with environmental noise color varied continuously from blue to white to red as shown in panel (a). Larger values of *α* represent higher intensities of competition from the invading species

Our analyses of the simulations of the Lotka–Volterra competition model (1) are summarized in Figure 6. Our focus was on the effects of the timescale of environmental fluctuations on the spectral properties of population numbers and the probability of extinction for the native species.

Figure 6a shows the protocol we used for the random environmental noise. We assumed a linear spectrum which varied from short timescale fluctuations (slope = 2, blue noise), to fluctuations with no autocorrelation (slope = 0, white noise), to long timescale fluctuations (slope = −2, red noise). The random time series generated by these spectra have the same mean of zero and same variance, the latter being proportional to the total area under the spectrum; they differ only in their timescale properties. For the simulations involving the computation of spectral exponents and extinction probabilities, we varied the spectral slope of the environmental noise in 101 small increments from +2 to −2, as indicated by the curved arrow in Figure 6a.

Figure 6b shows the population spectrum and cospectrum for the simulations involving blue noise, white noise, and red noise (Figure 6a). Since the parameter values for the two competing species are identical, and the properties of their environmental noise inputs are the same, the mean curves shown apply to both populations. As described in section 2.4, we used a cospectrum function that was equal to a constant fraction, 0.9, of the spectrum, so, for each color of environmental noise, the population spectrum and cospectrum are similar. For blue environmental noise, the smoothed normalized spectrum and cospectrum have low power at long timescales which increases and levels off at frequencies exceeding .1. This reflects the fact that, for the intrinsic rates of increase used in the simulations (*r*_1_ = *r*_2_ = .3), population growth is undercompensating, that is, perturbations from a stable equilibrium do not show damped oscillations in the deterministic version of the model. Previous work for single‐species population models has shown that undercompensating populations are sensitive to long timescale environmental noise, whereas overcompensating populations are sensitive to short timescale noise (e.g., Danielian, 2016). In effect, the slower response times of populations with small intrinsic rates of increase “filter out” the short timescale components of the environmental noise (Desharnais et al., 2018). This phenomenon can also be seen in the smoothed normalized spectrum and cospectrum for the populations subjected to white environmental noise. The population fluctuations are less sensitive to the shorter timescale components of the flat environmental spectrum producing a population spectrum and cospectrum that is biased toward long timescales (Figure 6b). Lastly, when the populations are subjected to environmental noise biased toward long timescales, the longer timescale components of the noise are enhanced and the shorter timescale components are suppressed, producing a smoothed normalized spectrum and cospectrum that is more strongly biased toward long timescales than the environmental noise (Figure 6b).

Figure 6c shows how the spectral exponents for the realizations of the population fluctuations and environmental noise change as the environmental noise is shifted gradually from blue, to white, to red (arrow in Figure 6a). Positive spectral exponents indicate spectra which are biased toward short timescales, and negative spectral exponents are indicative of long timescale fluctuations. Both the population and environmental spectral exponents decrease monotonically as the spectra for the environmental noise redden. However, the population spectral exponent starts out negative while the environmental spectrum is still strongly blue. As mentioned above, with the model parameter values used in our simulations, the dynamics of the two competing species acts as a “reddening filter,” producing population spectra that are more biased toward long timescales.

Of interest for conservation purposes is how the timescale of the fluctuations in the environmental noise influences the persistence of the native species. Figure 6d is based on simulations where an extinction threshold has been set arbitrarily to 5% of the carrying capacity. All other model parameter values are identical to the ones used for the simulations in Figure 6b,c. When the competition coefficients are equal (*α* = *β* = .25), the extinction probability for both species remains small until the color of the environmental noise begins to redden (Figure 6d). For the reddest environmental spectrum, both species have about a 55% probability of extinction. If the non‐native species has a competitive advantage, the influence of reddened environmental spectra on the persistence of the native species becomes more pronounced. Figure 6d shows how increasing the competition coefficient for the invading species to *α* = .50 and *α* = .75 increases the likelihood that the native species will be lost, while slightly lowering the extinction risk for the invasive species. For, *α* = .75, a reddening of the environmental spectrum quickly elevates the probability of extinction for the native species from a value of about 3% for the bluest environmental noise to a value which asymptotes at about 94% for the reddest environmental noise (Figure 6d). This suggests the possibility of a synergy between the effects of reddening environmental noise and competition from invasive species for the risk of extinction for native populations.

### 3.5 Climate data

**FIGURE 7:**
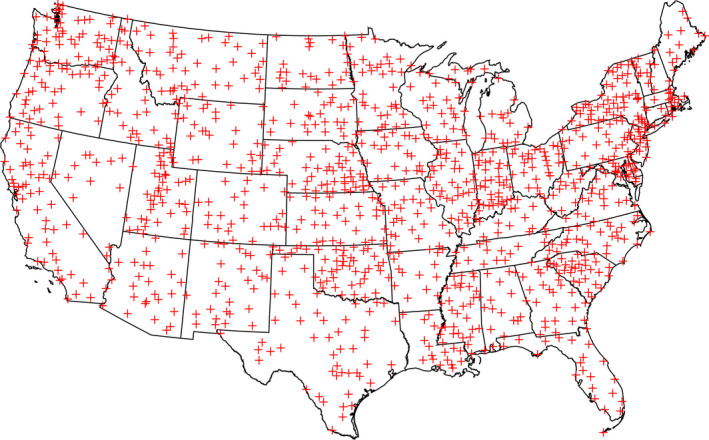
Geographic locations of the 1218 weather stations from which a 100‐year record (1915–2014) of mean annual temperatures were obtained. Data are from the U.S. Historical Climatology Network

We know that human impact on the climate system has resulted in an increasing trend of warming temperatures (Masson‐Delmotte et al., 2021). Given the observations and results of the previous sections, an important related question is whether there have been changes in the timescale of random environmental fluctuations around these trends. Our analyses make use of a 100‐year record (1915–2014) of mean annual temperatures from 1218 weather stations obtained from the U.S. Historical Climatology Network (Menne et al., 2009). Figure 7 shows the locations of the weather stations. Although not uniform in their distribution, they cover every state and region in the continental United States.

**FIGURE 8:**
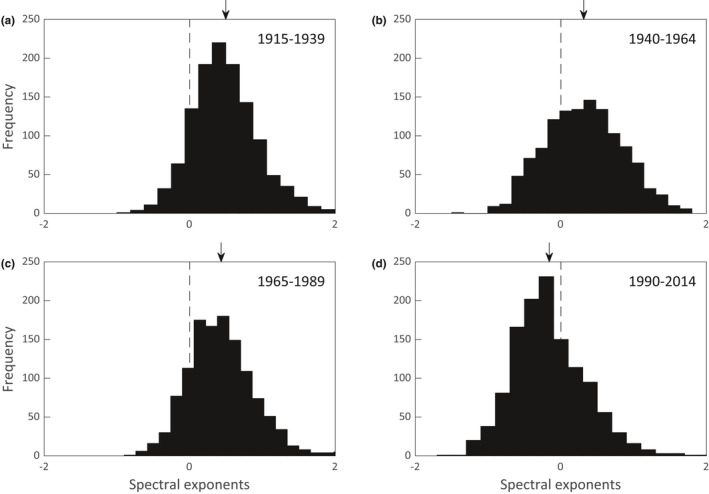
Spectral exponents for the 1218 time series of mean annual temperatures. Each 100‐year record was broken into four 25‐year intervals (a–d). The arrows indicate the locations of the mean values. The dashed lines represent a spectral exponent of zero. Positive spectral exponents indicate spectra biased towards short timescales (blue‐tinged noise) and negative values indicate a bias towards long timescales (red‐tinged noise)

To investigate evidence for change in the color of the mean annual temperature spectra over time, we divided the 100‐year record from each station into four 25‐year intervals and computed the spectral exponents for each time interval (see section 2.5). Figure 8 shows the histograms of spectral exponents for the 1218 stations. The dashed line represents the zero value (white noise environmental fluctuations); spectral exponents to the left indicate a red noise bias and those to the right represent a blue noise bias. The arrow at the top of each histogram shows the mean. The mean values are 0.500, 0.313, 0.432, and −0.160 for the range of years 1915–1939, 1940–1964, 1965–1989, and 1990–2015, respectively. It appears that there was a shift from 1990 to 2014 from blue‐shifted spectra to red‐shifted spectra. The significance values for the changes between adjacent time intervals are *p* = .046 for 1915–1939 vs. 1940–1964, *p* = .654 for 1940–1964 vs. 1965–1989, and *p* = 1.676 × 10^−10^ for 1965–1989 vs. 1990–2015.

**FIGURE 9:**
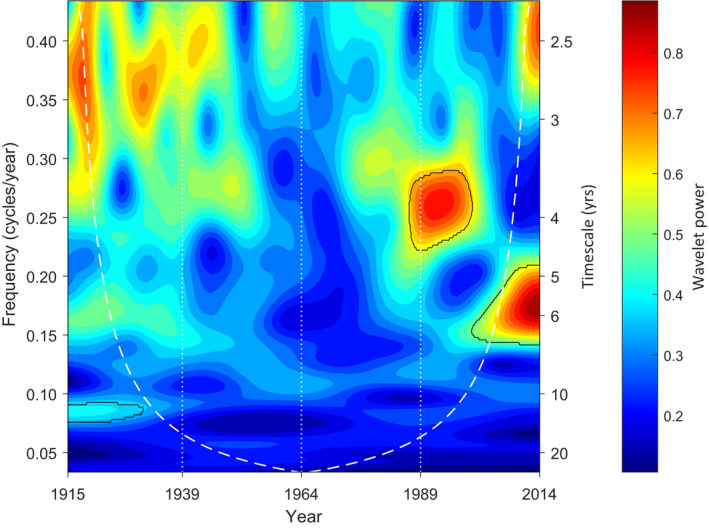
Mean field wavelet for 100‐year time series of mean annual temperatures. The wavelet is a visualization of how the timescale distribution of variation in mean annual temperatures has varied from 1915 to 2014. Each 100‐year time series was quadratically detrended and a Morse wavelet was obtained using the MATLAB default values of 3 and 60 for the symmetry and time‐bandwidth product, respectively. The wavelets for the 1218 stations were then averaged. The white dashed curve is the cone of influence where edge effects can affect the wavelet power. The dark curves indicate areas where the wavelet power is statistically significant at the 5% level, based on the upper 95th percentiles of 2000 surrogate data sets where the time series for all stations were reordered randomly in tandem. A change to longer timescale fluctuations is indicated by the significant shift in wavelet power to lower frequencies

The spectral analyses conducted for Figure 8 assume that the residual deviations from the fitted quadratic trends for each 25‐year time period are stationary, that is, the probability distribution and timescale properties of the residual time series are invariant. A mean field wavelet analysis which relaxes the stationarity assumption is presented in Figure 9 for the entire 100‐year time period. The regions of statistically significant wavelet power are outlined in black. They indicate that the timescale of the fluctuations in mean annual temperature, when averaged over all weather stations, has shifted to long timescale values of approximately 3.5–7 years for the period after 1980, again suggesting that there has been a recent reddening of the timescale for random fluctuations in mean annual temperatures around their changing trends.

## 4 DISCUSSION

Our cospectral correlation analysis does not imply a direct causal link between population numbers of the two species of tree squirrels and ambient temperature. There are many environmental and biotic factors which interact to impact the dynamics of natural populations. Although we used temperature as a proxy for environmental fluctuations, this metric is often associated with other environmental variables. For example, Zhao and Khalil (1993) have shown that mean monthly temperature and total monthly precipitation are negatively correlated in summer months over most of the contiguous United States. Excellent time series on temperatures are available and the data lend themselves to analysis by spectral methods. Time series of total monthly precipitation data, while available for the southwest Unite States, are not well‐suited for analysis using spectral methods, as they contain many consecutive values of zero.

Our spectral analyses of the WGS and FS census data suggest that most of the variation in animal numbers occurs on timescales that exceed 15 months. In the case of the FS, there is also evidence for variation on a six‐month timescale. This timescale‐specific variation may be due to changes in resource abundance, the timing and frequency of reproduction, and reproductive output.

Changes in population numbers on a long timescale could be due to variation in the supply of food resources on multi‐year, highly variable timescales. For example, acorns provide a valuable source of food for tree squirrels (Steele & Yi, 2020), but a very large (>600 g/m^2^) mast crop was only produced in one of the nine years in which we measured relative acorn production (Appendix B). We observed the production of a very large mast crop within our study area in the fall of 2013 (Table A2). Census counts for both species began to increase in the late spring and summer of 2013 and continued to increase through the spring of 2014 (Figure 2). A precipitous decrease in abundance was observed throughout the summer of 2014 which may have been brought about by dispersal of animals out of our study site. A very small acorn crop (<6 g/m^2^) was produced in the fall of 2014. A modest‐sized crop of acorns (~35 g/m^2^) produced in the fall of 2015 was followed by an increase in census counts for both species through the summer of 2016. Acorn production was very low in the fall of 2016, 2017, 2018, and 2019, and this long time period without a modest to large‐sized acorn crop corresponded to relatively low census counts for both species (≤ 20 animals). A modest acorn crop produced in the fall of 2020 again corresponded to an increase in census counts for both species during the summer and fall of 2020. Acorns are present in the trees for a prolonged period before they appear in significant quantities on the ground, so this food resource is also available to the animals prior to the fall of the year which may account for the high census counts in the summers prior to our acorn crop sampling periods.

Acorn production by coastal live oaks (*Quercus agrifolia*), a common tree species within our study area, is influenced by the amount of rain in the one or two years prior to the year in which acorns are produced (Koenig et al., 1996). Mann and Gleick (2015), as well as Diffenbaugh et al. (2015), documented that an increase in ambient temperatures has been accompanied by a decrease in rainfall within California. A plot of temperature and precipitation anomalies over the period of 1895 through November 2014 showed the 3‐year period ending in 2014 was by far the hottest and driest on record in California (Mann & Gleick, 2015). Diffenbaugh et al. (2015) documented that although there has not been a large change in the probability of either negative or moderately negative precipitation anomalies in recent decades, the occurrence of drought years has been greater in the two decades prior to their study than in the preceding century. In addition, the probability that precipitation deficits co‐occur with warm conditions and the probability that precipitation deficits produce drought have both increased. Climate model experiments by Diffenbaugh et al. (2015) revealed an increased probability that dry precipitation years are also warm years. Many regions of California were in moderate, severe, or extreme drought conditions for much of 2015 through 2021, except for most of 2019 (NCEI website, accessed 2 February 2022). So, the drought is long term and persisted for much of our study.

The yearly record of observations of juvenile and subadult individuals for both species shown in Appendix C illustrates the effect that long‐term variability of food resources may have on reproduction by the WGS and the FS over long timescales. As stated above, production of acorns varied widely between years and the production of other food supply items could certainly vary widely between years. Variability in the availability of food items each year along with changes in the number of juvenile and subadult animals could lead to population variability on long timescales, as observed in the spectral analysis of our data (Figure 3).

The availability of food in our study site also varied on a six‐month timescale. Items such as catkins from oak and walnut trees, flowers on *Fremontodendron* spp. and *Arctostaphylos* spp., and male cones on pine trees became available in the spring. Items such as acorns, walnuts, and fruit bodies from the California Bay Laurel (*Umbellularia californica*) and California Buckeye (*Aesculus californica*) became available in the fall of the year (Ortiz & Muchlinski, 2015). The timings (spring and fall) of the first availability of these food items on a yearly basis fit well with the potential timing of reproduction on a yearly basis by both the FS and WGS.

**FIGURE A1:**
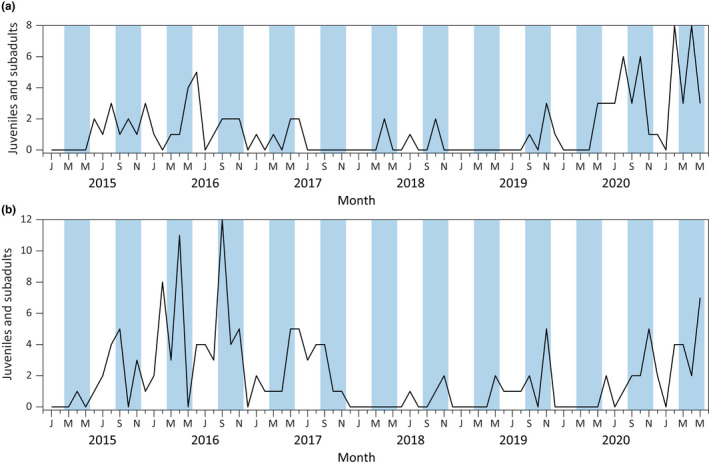
Monthly time series for numbers of juveniles and subadults for (a) the WGS and (b) the FS from January 2015 through May 2021. The shaded regions represent spring (March–May) and fall (September–November)

Two distinct periods of potential reproduction for the FS in Southern California were documented by King’s (2004) study of 135 L submitted to three wildlife rehabilitation centers during 2002. Approximately, 60% of litter production documented by King (2004) was associated with the months of February, March, and April, with the largest number of litters born in March. A second pulse of litter production occurred during the months of August, September, and October with the largest number of litters born in September, six months after the largest pulse of litters born during the spring. Although production of litters by the FS on a semi‐annual basis is possible, thus leading to an increase in observed population size on a semi‐annual basis, the number of juvenile/subadult animals observed during census counts in this study varied widely among years (Figure A1).

The WGS appears to exhibit a yearly pattern of reproduction different than the FS. Most research documents breeding activity in late fall and early winter months with birth of most litters in spring and early summer months (Carraway & Verts, 1994; King, 2004). A few pregnant females were observed in June, July, August, and September (Fletcher, 1963), and lactating females have been observed as late as October in Californian (Swift, 1977). However, no definite records of multiple pregnancies not attributable to intrauterine loss of the first litter are available (Bailey, 1936; Fletcher, 1963; Jameson & Peeters, 1988; Swift, 1977). The difference in reproductive patterns between the FS and the WGS could bring about the presence of a 6‐month cycle in abundance of the FS and the absence of a similar 6‐month cycle in the WGS. The difference in reproductive patterns could also give a competitive advantage to the FS in certain habitats through higher natality in years of good resource production.

Muchlinski et al. (2012) produced a Habitat Suitability Model (HSM) for the WGS and the FS which allowed short‐term and longer‐term coexistence habitats to be identified using a linear combination of three habitat variables: percent canopy cover, percent of deciduous trees, and average height of ground cover. Habitats with a low percentage of canopy cover, a high percentage of deciduous trees, and a low height of ground cover were classified as short‐term coexistence habitats. Locations with a high percentage of canopy coverage, a low percentage of deciduous trees, and a low height of ground cover were classified as longer‐term coexistence sites. (Sites with a high height of ground cover, a high percentage canopy cover, and a low percentage deciduous trees were identified as “exclusion habitats” where only the WGS is found, but the FS exists in adjacent habitats.) For example, Muchlinski et al. (2009) reported that the FS replaced the WGS in four years at a short‐term coexistence habitat, California State Polytechnic University, Pomona, which contained manicured and more natural areas on the campus with paved pathways and buildings surrounded by a mixture of *Juglans*, *Eucalyptus*, *Washingtonia*, *Pinus*, and other tree species. In contrast, the two species have coexisted within longer‐term coexistence habitats of Griffith Park in Los Angeles, CA, for more than 60 years, which were more natural in appearance consisting of *Pinus*, *Quercus*, *Umbellularia*, *Sequoia*, and *Ulmus* species, but with human‐influenced aspects such as picnic tables, a playground, and restrooms (DeMarco et al., 2020; King, 2004; King et al., 2010). The study area at CBG has been classified as a longer‐term coexistence habitat by Muchlinski et al. (2012). How long coexistence can continue in longer‐term coexistence habitats is unknown. Many longer‐term coexistence sites are fragments of habitat where the FS, but not the WGS, exists in surrounding habitats. The WGS is also subject to loss of genetic diversity in these habitat fragments as described by DeMarco et al. (2020).

The predictions of the competition model presented in section 3.4 can be interpreted in terms of the HSM developed by Muchlinski et al. (2012). The HSM implies that the competitive effects of the FS on the WGS are high in a short‐term coexistence site such as California State Polytechnic University, Pomona, and other former lowland coexistence sites (Cooper & Muchlinski, 2015). In terms of the competition model presented in Figure 6, the value of the competition coefficient *α* would be large relative to the coefficient *β*, and extinction of the WGS could occur under conditions of blue and red environmental noise. Conversely, a lower level of competition in a longer‐term coexistence site implies the values of *α* and *β* are more similar and a higher level of reddened environmental noise would be needed to bring about extinction of the WGS (Figure 6d). Our results from section 3.5 suggest that climate changes are increasing the timescale of yearly environmental fluctuations. Our spectral analyses of monthly census data suggest that most of the variation in numbers of the WGS and FS occurs over timescales of more than 15 months (Figure 2). Thus, aside from the effects of a warming climate, any changes in the timescale of temperature fluctuations around the increasing trend could represent an additional risk factor for the persistence of the WGS in some of its native range.

After the annual changes in mean monthly temperature were removed from the ONT data using a band‐stop filter, the remaining variation in temperature fluctuations was composed of a strong six‐month cycle and significant variation on timescales that exceeded 28 months (Figure 4c). Meteorologists and climate scientists have used harmonic analysis to document semi‐annual cycles in rainfall and temperatures whose amplitude and phase shift vary by geographical location, with moderate amplitudes for the southwest United States (Hsu & Wallace, 1976; White & Wallace, 1978). Analyzing North American temperature data from 1979 to 2018, North et al. (2021) used Bayesian analysis to fit a model with annual and semi‐annual harmonics that vary over space and time. They identify geographical regions with significant changes in the contributions of the two harmonics to seasonal cycles. In Appendix D, we used least squares to fit a model with annual and semi‐annual harmonics to the unfiltered mean monthly temperature data in Figure 4a and show that a model that includes both annual and semi‐annual cycles provides a significantly better fit to the data than a model based on the annual cycle alone. Figure 4c shows that the filtered temperature time series has the same period, phase, and approximate amplitude as the theoretical semi‐annual cycle. One can also see the effects of long timescale variability in the way the filtered data meanders above and below the semi‐annual cycle. The cospectra of Figure 5 indicate a significant negative correlation between the squirrel census data and the ONT filtered weather data at a timescale of six months. While we cannot demonstrate a direct causal mechanism for this correlation, this observation could motivate further research.

It was not possible to specify an estimated peak value for the timescale of low‐frequency variation in the squirrel numbers or mean monthly temperatures. The 140 months of the time series represent less than 12 years. In spectral analyses, estimates of long‐period, low‐frequency cycles are less precise since they cannot be as readily observed as short‐period, high‐frequency oscillations. In the estimated spectra of Figures 3 and 4, the spectral power continues to increase as the frequency decreases. However, the record of mean annual temperatures for Ontario Airport extends back to 1999, providing a 22‐year time series. In Appendix D, we show that a significant peak in the spectrum of annual temperatures occurs on a timescale of about 7 years, which is consistent with the wavelet analysis in Figure 9. If annual changes in environmental conditions are driving the long‐term variation in squirrel numbers, which seems to be the case for the WGS (Figure 5a), this estimate could also represent the timescale of those fluctuations.

We presented simulation results in section 3.4 that were designed to explore the effects of changes in the timescale of environmental noise on the outcome of competition between ecologically similar native and non‐native species. We showed that an increase in the timescale of environmental noise reddens the spectrum of population fluctuations and decreases the likelihood of coexistence, especially when the non‐native is a better competitor. This result differs from one of the findings of Ruokolainen and Fowler (2008), who concluded that extinction risk decreased with a reddening of environmental noise when, like in our model, there was a strong correlation in the species response to the environmental fluctuations. However, their simulation protocols differed from ours in several ways. First, they looked at a community of three competing species. Second, their environmental noise was generated using an autoregressive process and was added to the carrying capacity for each species. Third, and most importantly, in their models the intrinsic rate of increase for each species was set to *r_i_* = 1.8, whereas in our model we chose *r*_1_ = *r*_2_ = .3, which is more consistent with the reproductive capabilities of tree squirrels (Appendix A). In deterministic models of the type used in our simulations and those of Ruokolainen and Fowler (2008), values of 1 > *r* > 2 lead to overcompensating dynamics, where the approach to equilibrium exhibits damped oscillations. Although Ruokolainen and Fowler (2008) claimed that their results are qualitatively similar for values of *r* < 1, previous work with single‐species models (e.g., Danielian, 2016) has shown that extinction risk increases with a reddening of environmental noise when the deterministic model, like ours, has undercompensating dynamics (*r_i_* < 1), but decreases with a reddening of environmental noise when the deterministic model has overcompensating dynamics (1 < *r_i_* < 2). Since the apparent contradiction between our results and those of Ruokolainen and Fowler (2008) occurred when there was a strong synchrony among the three species due to the high correlation of the effects of environmental noise, their result is consistent with what one would expect from a single‐species model. When we repeated our simulations using values of *r*_1_ = *r*_2_ = 1.8, we observed a result consistent with Ruokolainen and Fowler (2008).

Our simulation results were limited in their scope. They were motivated by our analyses of timescale differences in the variability of population fluctuations for two sympatric species of tree squirrels. Simulation analyses of the type conducted in this paper could be expanded to include a broader examination of parameter space, other ecological interactions such as predator and prey, and larger communities of interacting species. Our approach could also be adapted to applied conservation models where environmental variability is a part of the simulation protocols. The inference in section 3.5 that timescale shifts in environmental fluctuations may be occurring due to climate change would be a motivation to explore the impacts for populations of interest to conservationists and natural resource managers.

Our analyses of spectral exponents for mean annual temperatures suggest that there has been a reddening of the timescale of climate fluctuations for the continental United States during the time period 1990–2014. García‐Carreras and Reuman (2011) conducted a global analysis of spectral exponents for the time period 1911–1990. They split the time series into two halves and concluded that, while most of the spectral exponents were red‐shifted, the red shift was smaller in 1951–1990 compared with 1911–1950. This was true for all continents except Asia, which was redder in 1951–1990 than it was in 1911–1950. Thus, in general, they observed a shift to shorter timescales in more recent times, while we observed the opposite. This inconsistency may be due to differences in our analyses. García‐Carreras and Reuman (2011) used a linear function to detrend their data, while we used a quadratic function. They divided their time series into two segments of 40 years each, while we divided ours into four segments of 25 years each. Most importantly, our last time series segment covered 1990–2014 which went beyond the latest year that they examined. It was in this last 25‐year period that we saw a strongly significant shift from blue‐tinged fluctuations to red‐tinged fluctuations (Figure 8). This agrees with the results of Wang and Dillon (2014) who also found an increase in the autocorrelation (timescale) of annual temperatures from the period of 1975 through 2013. It may be the case that the lengthening of the timescale of mean annual temperature fluctuations is a relatively recent phenomenon.

Our analyses of changes in climate fluctuations could be extended in several ways. The distributions in Figure 8 show that there is variation in the values of the spectral exponents among weather stations. The sampling locations, which are scattered across the continental United States (Figure 7), could be subdivided by region (e.g., northeast and southwest) to see whether there are significant differences in the values of the spectral exponents due to geographic location. Following García‐Carreras and Reuman (2011), our analyses could be expanded to include sampling stations on other continents and other measures, such as mean seasonal temperatures, could be analyzed. Total annual precipitation could also be included in the analyses. Further research is needed to see whether our inference that climate changes are lengthening the timescales for environmental fluctuations is robust. This could have implications, not only for conservation biology and resource management, but also for other areas such as forest fire management and agriculture.

## 5 CONCLUSIONS

Using spectral analyses, we have shown that the variations in monthly fluctuations of population numbers for native and non‐native tree squirrels coexisting in the same habitat are distributed mostly over long timescales (>15 months) and their numbers are synchronous over long timescales. After annual cycles are filtered from the time series for mean monthly temperatures from nearby Ontario Airport, there remains a strong six‐month cycle and significant fluctuations at timescales that exceed 15 months. There was a significant negative correlation between the temperature data and squirrel numbers for both WGS and FS at a six‐month timescale and a significant amount of long timescale correlation between mean monthly temperatures and WGS numbers. We used model simulations to show that environmentally induced long timescale variation in population numbers for two competing species with moderate rates of reproduction greatly increases the probability of extinction of the inferior competitor. Finally, we conducted spectral and wavelet analyses for 100 years of mean annual temperatures from 1218 weather stations across the continental United States. Our results suggest that the timescale of fluctuations around the changing climate trends has increased in the last few decades, providing another environmental aspect of climate change that could threaten the maintenance of biodiversity.

This study documents long timescale variation in natural populations of conservation interest, shows that long timescale variation can accelerate the loss of species diversity, and provides evidence that the timescales of environmental fluctuations have increased in recent times. We hope this serves as a cautionary message for conservationists and natural resource managers that an examination of timescales for environmental and population fluctuations are factors worthy of consideration.

## CONFLICT OF INTEREST

None declared.

## AUTHOR CONTRIBUTIONS

**Robert A. Desharnais:** Conceptualization (equal); Formal analysis (lead); Methodology (equal); Software (lead); Visualization (lead); Writing – original draft (lead); Writing – review & editing (equal). **Alan E. Muchlinski:** Conceptualization (equal); Data curation (lead); Investigation (equal); Methodology (equal); Project administration (lead); Supervision (lead); Writing – original draft (equal); Writing – review & editing (equal). **Janel L. Ortiz:** Investigation (equal); Writing – original draft (equal); Writing – review & editing (equal). **Ruby I. Alvidrez:** Investigation (equal); Writing – original draft (equal); Writing – review & editing (equal). **Brian P. Gatza:** Investigation (equal); Writing – review & editing (equal).

## Figures and Tables

**FIGURE A2:**
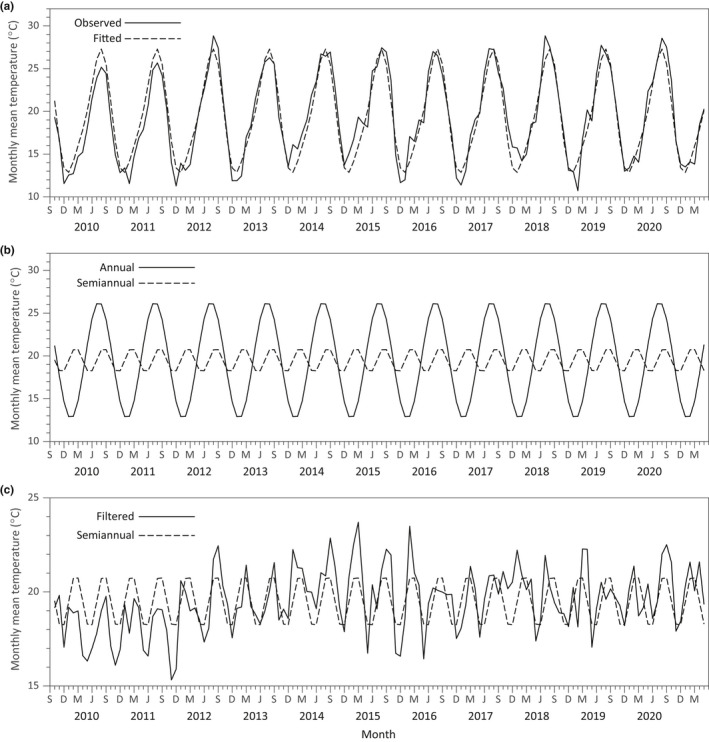
(a) Time series for observed and fitted mean monthly temperatures from Ontario Airport. (b) Plots of the component annual and semi‐annual cycles from the full model (equation A1). (c) Comparison plots of the filtered temperature time series and the theoretical semi‐annual cycle

**FIGURE A3:**
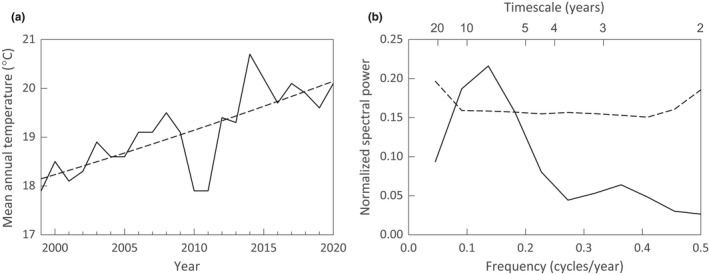
(a) Time series for observed mean annual temperatures from Ontario Airport. The dashed line is the fitted quadratic trend curve which is nearly linear. (b) Smoothed normalized spectrum for the standardized residuals from the temperature time series in panel (a). Dashed line is the 95% significance threshold for the null hypothesis of no timescale dependence in the ordering of the residuals

## Data Availability

Squirrel census data are archived on Dryad (https://doi.org/10.5061/dryad.w6m905qqv). Weather and climate data are accessible at the Climate Data Online website of NOAA’s National Centers for Environmental Information (https://www.ncdc.noaa.gov/cdo‐web/). MATLAB code used for computing and smoothing the spectra and cospectra and generating univariate or multivariate time series with the asymptotic spectral properties of a supplied spectral matrix is available online at: https://doi.org/10.5281/zenodo.5753478.

## APPENDIX A

### Intrinsic rates of increase for squirrels

We used the Euler‐Lotka equation to compute the intrinsic rate of increase, *r*, for the *Sciurus* species in table 1 of Wood et al. (2007). For this equation, *r* is the solution to

(A1)
$$\sum_{x=0}^{\infty }e^{‐rx}l_{x}b_{x}=1$$

where *l_x_* is the survival probability to age *x* and *b_x_* is the number of offspring born to an individual of age *x*. In their population viability analysis of tree squirrels, Wood et al. (2007) give values for the percentage of females breeding per season, *P*, the number of breedings per year, *β*, the litter size per female, *L*, and the mortality rates for age zero, *m*_0_, and individuals one year and older, *m* (Table A1). For each species, breeding begins at age one. Given this information, we computed annual survival rates as *l*_1_ = 1–*m*_0_ and *l_x_* = (1–*m*_0_)(1–*m*)*^x^* ^–1^ for *x* ≥ 2. (By definition, *l*_0_ = 1.) The number of offspring per individual was computed as *b*_0_ = 0 and *b_x_* = *PβL*/2 for *x* ≥ 1. We divide by 2 because only females produce offspring and only half the litter are female. The Euler‐Lotka equation becomes

$$\begin{array}{c} \sum_{x=1}^{\infty }\left(P\beta L/2\right)\left(1‐m_{0}\right){\left(1‐m\right)}^{x‐1}e^{‐rx}=\left(P\beta L/2\right)\left(1‐m_{0}\right)e^{‐r}\sum_{x=0}^{\infty }{\left[\left(1‐m\right)e^{‐r}\right]}^{x}\\ =\left(P\beta L/2\right)\left(1‐m_{0}\right)e^{‐r}{\left(1‐\left(1‐m\right)e^{‐r}\right)}^{‐1}=1. \end{array}$$

Taking logarithms, we get.

(A2)
$$\ln\left[\left(P\beta L/2\right)\left(1‐m_{0}\right)\right]‐r‐\ln\left[1‐\left(1‐m\right)e^{‐r}\right]=0$$

A root‐finding algorithm was used to numerically solve equation A2 for *r*.

Wood et al. (2007) provided three sets of the life history values described for the following species: *Sciurus aberti*, *S*. *carolinensis*, *S*. *granatensis*, *S*. *niger* (FS), and *S*. *vulgaris*. The three sets of parameters are labeled as “optimistic,” “average,” and “pessimistic.” Using the values labeled as average, we computed the values of *r*, respectively, for the five species (Table A1). The average of these five values is 0.32. Therefore, we used a value of *r*_1_ =* r*_2_ = .3 in our simulations. This is close to the value of 0.29 obtained for FS (Table A1).

## APPENDIX B

### Numbers of acorns

Estimates of acorn production were made in years 2012 through 2020 by measuring during the middle of October the mass of acorns in a one square meter plot under each of six oak trees within the study area. The same trees and the same plots were used each year for estimation of acorn production to assess variability among years and relationship to changes in abundance of the tree squirrels. The data appear in Table A2.

## APPENDIX C

### Numbers of juvenile and subadult squirrels

Starting in January 2015, observers at our field site started distinguishing among adults, subadults, and juveniles. The latter two categories can serve as a proxy for reproduction. Figure A1 shows the numbers of juveniles and subadults for the WGS and the FS. For both species, juveniles and subadults are seen more often in spring and fall (Figure A1, shaded areas), especially in the case of the FS. However, there is a great deal of year‐to‐year variation (long timescales) in the numbers.

## APPENDIX D

### Annual and semi‐annual temperature cycles

Meteorologists and climatologists have used harmonic analysis to identify seasonal cycles in atmospheric temperature (White & Wallace, 1978). In addition to a strong annual cycle, a semi‐annual cycle, which can vary by year and location, has been identified (North et al., 2021; White & Wallace, 1978). In their equation (1), North et al. (2021) used the first two terms of a Fourier representation of an annual temperature time series. Their equation, using months as the time unit, is given by

(A3)
$$x_{t}=a_{0}+A_{1}\cos\left(\frac{2\pi \left(t+\phi_{1}\right)}{12}\right)+A_{2}\cos\left(\frac{4\pi \left(t+\phi_{2}\right)}{12}\right),$$

where *x_t_* is the temperature (in °C), *t* is the time (in months), *a*_0_ is the center value for the temperature oscillations (in °C), *A_i_* is the amplitude (in °C) and *ϕ_i_* is the phase shift (in months) of the annual (*i *= 1) and semi‐annual (*i *= 2) component cycles.

We fit equation (A3) to the mean monthly temperature data from Ontario Airport (ONT) (Figure 4a) using the method of nonlinear least squares. The parameter estimates and their standard errors appear in Table A3. The phase shifts are relative to the month of September. The temperature data and the fitted function appear in panel A of Figure A2. The fitted function provides a good description of the observed temperature time series.

The two component cycles are shown separately in panel B of Figure A2. The annual cycle is the larger of the two and is obtained by setting *A*_2_ = 0 in equation (A3). It peaks in July–August and has its trough in January–February. The smaller semi‐annual cycle, obtained by setting *A*_1_ = 0 in equation (A3), peaks twice per year in February–March and August–September and has its troughs in November–December and May–June. The amplitude of the semi‐annual cycle is 21% the size of the amplitude of the annual cycle.

In panel (c) of Figure A2, we plotted the temperature time series obtained after filtering out the annual cycle (Figure 4a, dashed line) with the semi‐annual cycle from panel (b) of Figure A2. The theoretical and filtered cycles have the same periodicity, phase, and approximate amplitude. Moreover, one can see the effects of long timescale variability in the way the filtered data meanders above and below the semi‐annual cycle. This gives us confidence that the band‐stop filter used to attenuate the annual cycle worked well.

We fit equation (A3) to the observed temperature time series with *A*_2_ = 0, so that only the annual cycle was present. The parameter estimates and their standard errors are also presented in Table A3. We computed the following Akaike Information Criterion (AIC) for both fitted models:

$$AIC=n\ln\left(\frac{SS_{E}}{n}\right)+2k,$$

where *n* = 140 is the sample size, *SS_E_* is the residual sum of squares for the regression, and *k* is the number of fitted parameters (*k* = 3 for the annual model and *k* = 5 for the model with both annual and semi‐annual cycles). The annual model had an AIC of 165.02 and the model with both annual and semi‐annual cycles had an AIC of 114.82. The smaller AIC suggests that the model with both cyclic components is a better description of the seasonal temperature changes. The magnitude of the difference, ΔAIC = 50.20, suggests that the annual model has little support relative to the full model.

## APPENDIX E

### Mean annual temperature data for Ontario airport

We conducted a spectral analysis of mean annual temperatures for Ontario Airport (ONT). The data values are the averages of the 12 mean monthly temperatures for each year. The first year of complete data is for 1999, so the time series spans 22 years from 1999 through 2021 (Figure A3, panel (a)). We used the same methods as described in section 2.5 for climate data: we detrended the data by fitting a quadratic polynomial using least squares, computed the standardized residuals, and computed a smoothed normalized spectrum for the residual time series. Because the time series is relatively short, we used smaller spans of 3 and 3 data points for the two iterations of the smoothing algorithm. We computed 95% significance threshold by generating 2000 random permutations of the residuals, obtaining a smoothed normalized spectrum for each, and using the 95th percentiles of these surrogate spectra for the 95% limits. Figure A3 shows the ONT temperature data and spectrum. The fitted quadratic polynomial (dashed line in Figure A3, panel (a)) is nearly linear and suggests a trend of increasing temperatures. Although the significance band is wide due to the short length of the time series, the spectrum for the residuals shows a significant peak at a timescale of about 7 years (Figure A3, panel (b)). This corresponds to the timescale range of the local peak in mean wavelet power in the lower right corner of Figure 9.

**TABLE A1:** Parameter estimates from Wood et al. (2007, table 1, “average”) for five species of tree squirrels from the genus *Sciurus*

Parameter	Description	*Sciurus aberti*	*Sciurus carolinensis*	*Sciurus granatensis*	*Sciurus niger*	*Sciurus vulgaris*
*P*	% females breeding	62	70	70	55	90
*β*	breedings per year	2	2	2	2	2
*L*	litter size (per female)	3.4	2.8	2.2	2.2	2.2
*m*_0_	mortality (1st year)	0.60	0.60	0.60	0.60	0.62
*m*	mortality (≥1 year)	0.28	0.338	0.48	0.28	0.32
*r*	rate of increase (year^–1^)	0.45	0.37	0.13	0.29	0.36

**TABLE A2:** Mass of acorns (g) in a 1 m^2^ plot under each tree in the study area

Tree	Year								
	2012	2013	2014	2015	2016	2017	2018	2019	2020
Blue oak	0	250	6	83	0	7	12	0	98
Canyon oak	230	960	0	53	54	19	27	84	68
Coast live oak	18	290	18	29	3	5	3	0	5
Coast live oak	75	880	6	0	2	4	0	1	16
Coast live oak	69	590	2	34	39	4	0	0	24
Coast live oak	2	680	2	10	14	0	0	0	136
Mean ± SE	65.7 ± 35.5	608.3 ± 120.1	5.7 ± 2.7	34.8 ± 12.3	18.7 ± 9.2	6.5 ± 2.7	7.0 ± 4.4	14.2 ± 14.0	57.8 ± 21.2

**TABLE A3:** Parameter estimates and standard errors for full and annual models

Parameter	Description	Estimate (±SE) for full model	Estimate (±SE) for annual model
*a*_0_	center value of cycles (°C)	19.50 ± 0.12	19.49 ± 0.15
*A*_1_	amplitude of annual cycle (°C)	6.82 ± 0.18	6.80 ± 0.21
*ϕ*_1_	phase of annual cycle (mo.)	1.52 ± 0.05	1.53 ± 0.06
*A*_2_	amplitude of semi‐annual cycle (°C)	1.42 ± 0.18	—
*ϕ*_2_	phase shift of semi‐annual cycle (mo.)	0.48 ± 0.12	—
